# Psychometrics and diagnostics of the Italian version of the Alternate Verbal Fluency Battery (AVFB) in non-demented Parkinson’s disease patients

**DOI:** 10.1007/s10072-024-07436-5

**Published:** 2024-03-11

**Authors:** Edoardo Nicolò Aiello, Francesca Mameli, Fabiana Ruggiero, Eleonora Zirone, Stefano Zago, Sylvie Piacentini, Barbara Poletti, Maria Rita Reitano, Gabriella Santangelo, Nicola Ticozzi, Vincenzo Silani, Alberto Priori, Roberta Ferrucci

**Affiliations:** 1 Department of Neurology and Laboratory of Neuroscience, IRCCS Istituto Auxologico Italiano, Milan, Italy; 2 Fondazione IRCCS Ca’ Granda Ospedale Maggiore Policlinico, Milan, Italy; 3 Fondazione IRCCS Istituto Neurologico Carlo Besta, Milan, Italy; 4 Department of Oncology and Hemato-Oncology, Università Degli Studi Di Milano, Via Santa Sofia 9, 20122 Milan, Italy; 5 ASST Santi Paolo e Carlo, San Paolo University Hospital, Milan, Italy; 6 Department of Psychology, University of Campania “Luigi Vanvitelli”, Caserta, Italy; 7 Department of Pathophysiology and Transplantation, “Dino Ferrari” Center, Università degli Studi di Milano, Milan, Italy; 8 “Aldo Ravelli” Center for Neurotechnology and Experimental Brain Therapeutics, Department of Health Sciences, University of Milan, Milan, Italy

**Keywords:** Verbal fluency, Parkinson’s disease, Language; Executive, Neuropsychology, Cognitive impairment

## Abstract

**Background:**

Verbal fluency (VF) tasks are known as suitable for detecting cognitive impairment (CI) in Parkinson’s disease (PD). This study thus aimed to evaluate the psychometrics and diagnostics of the Alternate Verbal Fluency Battery (AVFB) by Costa et al. (2014) in an Italian cohort of non-demented PD patients, as well as to derive disease-specific cut-offs for it.

**Methods:**

*N* = 192 non-demented PD patients were screened with the Montreal Cognitive Assessment (MoCA) and underwent the AVFB—which includes phonemic, semantic and alternate VF tests (PVF; SVF; AVF), as well as a Composite Shifting Index (CSI) reflecting the “cost” of shifting from a single- to a double-cued VF task. Construct validity and diagnostics were assessed for each AVFB measure against the MoCA. Internal reliability and factorial validity were also tested.

**Results:**

The MoCA proved to be strongly associated with PVF, SVF and AVF scores, whilst moderately with the CSI. The AVFB was internally consistent and underpinned by a single component; however, an improvement in both internal reliability and fit to its factorial structure was observed when dropping the CSI. Demographically adjusted scores on PVF, SVF and AVF tests were diagnostically sound in detecting MoCA-defined cognitive impairment, whilst this was not true for the CSI. Disease-specific cut-offs for PVF, SVF and AVF tests were derived.

**Discussion:**

In conclusion, PVF, SVF and AVF tests are reliable, valid and diagnostically sound instruments to detect cognitive impairment in non-demented PD patients and are therefore recommended for use in clinical practice and research.

## Background

Up to 40% of non-demented patients with Parkinson’s disease (PD) present with dysexecutive-like, widespread cognitive impairment (CI) [1], which adversely affects their functional outcomes [2], prognosis [3, 4] and survival [5]. Therefore, the early detection of CI via clinimetrically sound tests is clinically crucial in this population [6].

Verbal fluency (VF) tests have been systematically found to be appropriate for this goal [7], as they capture both dysexecutive-inattentive features and lexical-semantic deficits that characterize PD [8] also in the early stages [9–11]. Indeed, in this population, VF measures have been successfully linked to those brain networks supporting both executive functions and language both in vivo [12–17] and at a neuropathological level [18]. Consistently, their utility has been proven either as individual screeners [9] or when included within second-level cognitive batteries [6]. Remarkably, VF measures have been also shown to be associated with patients’ motor and functional outcomes [19–22] and are acknowledged as sensitive indices of post-deep brain stimulation CI [23]. In addition, since VF tests are brief and require only verbal responses, they are suitable for fatigable patients and they are not affected by upper-limb disabilities, making them highly feasible in PD [24].

As highlighted by the Movement Disorders Society (MDS) [25, 26], there is a need for disease-specific clinimetric studies that address those tests that have been historically shown to be appropriate for detecting CI in PD, as is the case for VF. Such investigations would increase their level of recommendation for use in clinical practice and research [27]. Indeed, after a given test is made available to the clinical and scientific community and standardized in the normotypical population, clinimetric evidence in target patient cohorts should be always provided in order to improve users’ confidence in employing that test in real life, either clinical or research settings [27]. Additionally, with specific regards to test norms, it has been shown that cut-offs derived in normotypical populations—*i.e.*, normality thresholds—might be poorly sensitive and, at variance, disproportionately biased towards specificity [28, 29]. Due to the detrimental entailments of such a stance towards clinical and research practice, researchers in the field of cognitive testing often commit to provide users with disease-specific cut-offs [28–37]—at least with regard to those brain disorders that are among the most prevalent and incident, as is the case for PD [35–39].

As to the Italian scenario, only two studies by Biundo et al. [38, 39] have to this day focused on the clinimetrics of VF tests in PD patients and have shown that both phonemic and semantic VF tests (PVF; SVF) are diagnostically sound for detecting CI in this population. However, these studies [38, 39] referred to an outdated normative dataset—*i.e.*, that delivered by Novelli et al*.* [40] in 1986—and, most unfortunately, preceded the availability of the Alternate Verbal Fluency Battery (AVFB), standardized by Costa et al. [41] in 2013. Indeed, Costa et al*.* [41] not only provided updated norms for PVF and SVF test, but also normed, for the first time in Italy, an alternate phonemic-semantic VF task (AVF) —whose clinical utility in PD has been known for decades [42–44] and was recently demonstrated in Italy too [24]. AVF tests in fact represent a suitable alternative to much more common measures of set-shifting abilities that are, however, biased by PD patients’ motor disabilities—such as the Trail-Making Test-B [44]. This stance also happens to be supported by the fact that AVF tasks have been included in PD-specific cognitive screening batteries—such as the Parkinson Neuropsychometric Dementia Assessment [43] and the Parkinson’s Disease Cognitive Rating Scale [44].

Given the above premises, the primary scope of this study was to assess the construct validity, factor structure and internal consistency of Costa et al.’s [41] AVFB, as well as to examine its diagnostic properties, in an Italian cohort of non-demented PD patients. Moreover, the current investigation was aimed at deriving, from the abovementioned patient cohort, disease-specific cut-offs for Costa et al.’s [41] AVFB that could be used by Italian practitioners and clinical researchers.

## Methods

### Participants

Data on *N* = 192 Italian-speaking PD patients were retrospectively collected (126 males and 66 females; mean age = 62.6 ± 9.8 years; mean education = 12.1 ± 4.1 years). Patients were diagnosed with idiopathic PD (disease duration *range* = 3–20 months), between 2018 and 2023, according to the UK Brain Bank Criteria [45] by a team of expert neurologists through anamnestic interviews, neurological assessments, neuroradiological examinations and neuropsychological testing. Subjects were on medication during the cognitive assessment. Patients were free of PD-unrelated psychiatric disorders as well as of dementia—according to DSM-V criteria for a major neurocognitive disorder due to PD [46].

### Materials

Patients were assessed with the AVFB by Costa et al. [41], which includes three subtests, namely the PVF, the SVF and the AVF. Cues for the PVF were the letters F, A and S, whilst those for the SVF were colors, animals and fruits. The AVF requires examinees to continuously alternate letter and category-cued words as follows: A—colors; F—animals; and S—fruits. In all of the above subtests, patients were given 60 s for each trial and instructed not to produce proper nouns, place names, numbers, or inflected words with the same suffix. The order of presentation was the following: (1) PVF, (2) SVF and (3) AVF. A Composite Shifting Index (CSI) reflecting the cost of switching from a single-cued VF task to a double-cued VF task was then calculated as follows: AVF/[(PVF + SVF)/2].

In addition, for clinical purposes, patients were assessed using either the Mini-Mental State Examination (MMSE) [47] or the Montreal Cognitive Assessment (MoCA) [48]. Because the vast majority of patients were administered the MoCA (73.4%), those who underwent the MMSE (*N* = 53) had their MMSE scores converted into MoCA ones via the equating algorithm by Aiello et al. [49]. Such a conversion proved to be fully valid, since the same two patients who scored below the cut-off on the MMSE [47] were also classified as impaired by the derived MoCA scores [48], with no further discrepancies being noted (Cohen’s *k* = 1; *p* < 0.001).

### Statistics

#### Outcome measure

In the context of construct validity and diagnostic analyses, the MoCA was considered as an outcome, both in accordance with the MDS recommendations to use this test for screening purposes in this population [50] and given that the MoCA is widely known to highly load on executive-based cognitive processes [48], as is the case with VF tests [51]. In support to such an approach, the MoCA has already been used earlier as an outcome measure for testing the validity and diagnostics of executive-loaded tests in both normotypical [48, 52] and extrapyramidal populations [31, 35].

#### Psychometrics

Both AVFB and MoCA scores are normally distributed, as indexed by skewness and kurtosis values <|1| and |3|, respectively [53]. Hence, the construct validity of each VFB measures was tested against the MoCA via Bonferroni-corrected Pearson’s correlation coefficients. The effect size of correlation coefficients was classified as follows: (1) 0.10 < *r*_*s*_ ≤ 0.30 → small; (2) 0.30 < *r*_*s*_ ≤ 0.50 → medium; (3) *r*_*s*_ > 0.50 → large [54]. Internal reliability and factorial validity of the AVFB were assessed via Cronbach’s *α* coefficient and a principal component analysis (PCA), respectively, by addressing all of its subtests—*i.e.*, PVF, SVF, AVF and CSI scores.

#### Diagnostics

In order to test the diagnostics of each AVFB subtests, receiver-operating characteristic (ROC) analyses were carried out by operationalizing the positive state as an age- and education-adjusted MoCA score below or equal to the inner tolerance limit of the current Italian normative dataset [48]. Indeed, this threshold gives a safety level of at least 95% that least 95% of the normotypical population works beyond it [55, 56]. For those subtests yielding an acceptable AUC value (*i.e.*, ≥ 0.70) [57], sensitivity, specificity and positive/negative likelihood ratios were then calculated at the optimal cut-off value identified by means of the Youden’s index*.* Positive likelihood ratio values ≥ 2 and negative likelihood ratio ones ≤ 0.5 were deemed as optimal [58]. Standardized positive/negative predictive values [58] were calculated by assuming that the prevalence of cognitive impairment in the PD population is 40% based on recent meta-analytical evidence [1]. The Number Needed for Screening Utility (NNSU) was then computed—with values ≤ 1.02 meaning that less than ≈1 individual needs to be screened for the test to be useful in the view of ruling-in/ruling-out the presence of the target condition [59]. For the purpose of these analyses, PVF, SVF, AVF and CSI scores were adjusted for significant demographic confounders based on Costa et al.’s [41] normative dataset.

#### Software

Analyses were carried out with IBM® SPSS® 27, R 4.3 (https://cran.r-project.org/) and jamovi 2.3 (https://www.jamovi.org/). The significance threshold was set at = 0.05 and Bonferroni-corrected whenever necessary.

## Results

Table 1 summarizes patients’ demographic and cognitive measures.

**Table 1:** Patients’ demographic and cognitive measures

***N***	192
**Sex (male/female)**	126/66
**Age (years)**	62.6 ± 9.8 (37–85)
**Education (years)**	12.1 ± 4.1 (5–19)
**MoCA**	24.7 ± 3.4 (13–30)
Below-iTL proportion (%)^a^	10%
**PVF**	35.8 ± 12.7 (6–78)
**SVF**	42.4 ± 10.1 (19–77)
**AVF**	26.7 ± 12.9 (2–72)
**CSI**	0.7 ± 0.2 (0.1–1.2)

*MoCA* Montreal Cognitive Assessment, *PVF* phonemic verbal fluency, *SVF* semantic verbal fluency, *AVF* alternate verbal fluency, *CSI* Composite Shifting Index

^a^Aiello et al*.*’s [48] normative dataset

### Psychometrics

At *α*_adjusted_ = 0.013, MoCA scores proved to be strongly associated with PVF (*r*(192) = 0.53; *p* < 0.001), SVF (*r*(192) = 0.51; *p* < 0.001) and AVF (*r*(192) = 0.54; *p* < 0.001) scores, whilst moderately with the CSI (*r*(192) = 0.34; *p* < 0.001).

The AVFB was internally reliable (Cronbach’s *α* = 0.77), with item-rest correlation coefficients ranging from 0.49 to 0.76; however, a nine-point increase in reliability could be obtained by dropping the CSI (Cronbach’s *α* = 0.86)—with the same not being true for remaining measures. Consistently, the PCA yielded a mono-component structure accounting for 67.65% of the variance (loading *range* = 0.67–0.97). However, when the CSI was exploratively dropped from the PCA, the AVFB retained its mono-component structure, although an increase in both explained variance (79.44%) and average loading size (*range* = 0.0.88–0.90) was detected.

### Diagnostics

**Fig. 1:**
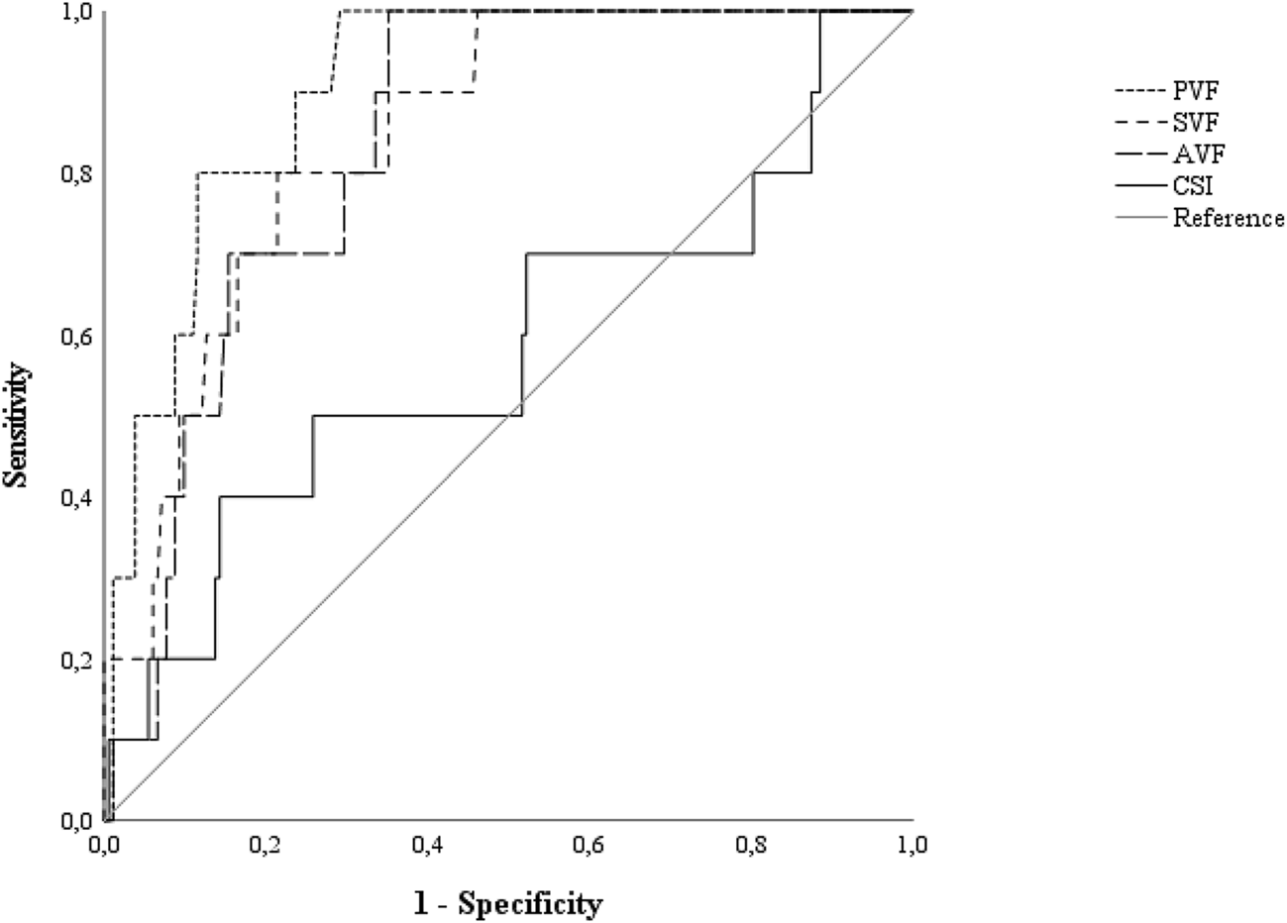
ROC curves for demographically adjusted AVFB measures. ROC, receiver-operating characteristics; AVFB, Alternate Verbal Fluency Battery; PVF, Phonemic Verbal Fluency; SVF, Semantic Verbal Fluency; AVF, Alternate Verbal Fluency; CSI, Composite Shifting Index. PVF: AUC = 0.91; *SE* = 0.03; CI 95% [0.84, 0.97]; SVF: AUC = 0.85; *SE* = 0.05; CI 95% [0.75, 0.94]; AVF: AUC = 0.84; *SE* = 0.04; CI 95% [0.76, 0.92]; CSI: AUC = 0.58; *SE* = 0.11; CI 95% [0.37, 0.79]. AVFB measures were demographically adjusted according to Costa et al*.* [41]

Ten out of 192 patients (5.2%) performed below the inner tolerance limit on the MoCA. In identifying such patients, adjusted PVF, SVF and AVF scores were highly accurate, whilst the CSI yielded an unacceptable AUC value (Fig. 1). Disease-specific cut-offs and associated diagnostic metrics were therefore only calculated for PVF, SVF and AVF tests, resulting overall optimal (Table 2). According to these thresholds, 33% of the sample was classified as impaired on the PVF, 24% on the SVF and 39% on the AVF.

**Table 2:** Cutoffs and associated diagnostics metrics for demographically adjusted AVFB measures

	Cutoff	*J*	Se	Sp	SPPV	SNPV	LR +	LR-	NNSU
PVF	< 29.537	0.70	1	0.71	0.70	1	3.43	0	0.71
SVF	< 38.038	0.59	0.80	0.79	0.71	0.85	3.73	0.26	0.81
AVF	< 25.191	0.65	1	0.65	0.65	1	2.84	0	0.80

AVFB measures were demographically adjusted according to Costa et al*.* [41]

*AVFB* Alternate Verbal Fluency Battery, *PVF* Phonemic Verbal Fluency, *SVF* Semantic Verbal Fluency, *AVF* Alternate Verbal Fluency, *Se* sensitivity, *Sp* specificity, *SPPV* standardized positive predictive value, *SNPV* standardized negative predictive value, *LR* + positive likelihood ratio, *LR-* negative likelihood ratio, *NNSU* Number Needed for Screening Utility

## Discussion

The present study provides Italian practitioners and clinical researchers with relevant psychometric and diagnostic information on Costa et al*.*’s [41] AVFB in non-demented PD patients.

In the current study, the AVFB proved to be internally consistent and valid at both the factorial and construct levels. However, it was found that by removing the CSI from the VFB, there was an improvement in its internal reliability as well as the extent to which the VFB itself fitted the underlying factor structure. Consistently, albeit significant, the correlation between the CSI and the MoCA was found to be weaker than that between the other AVFB subtests and the MoCA itself. Such results, together with the fact that the CSI, unlike the other AVFB subtests, was both able to distinguish PD patients with CI from those without CI, suggest that this measure has little or no clinical utility in this population.

Importantly, this report demonstrates the diagnostic value of PVF, SVF and AVF tests in non-demented PD patients. As to the PVF and the SVF, the present results align with the relevant literature by further confirming the usefulness of these tests for detecting CI in this population [24; 38; 39]. Additionally, it has been herewith shown, for the first time in Italy, that the AVF too is a diagnostically sound test to the abovementioned aim. This is consistent with previous reports supporting the use of the AVF as a motor-free measure of set-shifting abilities in PD [24, 42–44].

Moreover, the abovementioned findings are consistent with earlier neuroradiological reports showing, in this population, an association between VF performances and both the involvement of striatal structures [14, 16]—*i.e.*, the neural hallmark of PD—and the integrity of networks supporting language [12, 15, 17] and attentive-executive processes [44].

In addition, the present report supports the notion that VF tests are appropriate screening tools in this population [10], as evidenced by the fact that PVF, SVF and AVF tests were characterized by optimal NNSU values.

Relevantly, the current study provides Italian clinicians and researchers with disease-specific cut-offs for PVF, SVF and AVF tests, which can be used for detecting CI after adjusting the raw scores on such tests for significant demographic confounders on the basis of Costa et al*.*’s [41] normative dataset.

Of course, this study is not free from limitations. First, due to its retrospective nature, no functional or motor data could be retrieved. Therefore, it was not possible to assess the extent to which such variables might have affected VF performance. Such an issue is most evident when referred to dysarthric features which, as already outlined in the Italian scenario, could bias the results of timed tasks requiring verbal responses [60].

To account for this issue, a promising tool might lie in the Verbal Fluency Index (VFI), a measure originally developed to control for the confounding effect of dysarthria on VF performance in amyotrophic lateral sclerosis (ALS) [61]. Since the VFI has been recently normed for the Italian population and evaluated for its clinimetrics in ALS [62], it is recommended that future studies attempt to investigate its feasibility in PD as well. Second, it should be noted that the prevalence of CI in the current cohort was particularly low (*i.e.*, 5.2%) than that proposed in a recent meta-analysis on the subject (*i.e.*, 40%) [1]. Therefore, further research aimed at confirming the diagnostic power of the AVFB in a more balanced PD cohort is needed. Relatedly, the present reports did not include demented patients, who should be the focus of future investigations on the AVFB. Third, since this study was retrospective, it only assessed the reliability of the VFB at an internal level. Therefore, future studies should also focus on examining its inter-rater and test–retest reliability. Fourth, the target condition was herewith operationalized using a first-level test—*i.e.*, the MoCA: in order to be able to continue to use the current state of knowledge, it is desirable that future reports also use extensive second-level cognition to diagnose AFB examine battery.

In addition to the above, it makes sense to mention further, practically relevant information for future investigations. First, repeated-measure studies that explore the longitudinal feasibility of VFB in PD are still needed. This also includes the derivation of thresholds for defining clinically significant changes. Such investigations would be of great interest for at least two reasons: first, because VF tests are known to be able to detect involutionary trends in cognition after deep brain stimulation surgery [23]; second, because such measures have proved promising in predicting the onset of dementia in this population [63]. Furthermore, given the clear benefits that remote cognitive testing can offer when referred to patients with motor disabilities [64], it is desirable that future studies assess the clinimetrics and feasibility of the telephone-based version of Costa et al.’s [41] AVFB, which has been recently standardized for the Italian population [65].

In conclusion, the present study confirms that Costa et al*.*’s [41] AVFB, and more specifically its PVF, SVF and AVF subtests, is reliable, valid, and diagnostically sound instruments to detect CI in non-demented PD patients. Their use is therefore recommended in clinical practice and research.

## Data Availability

Datasets related to the present study are available upon reasonable request from interested researchers.
